# Mapping mutations in plant genomes with the user-friendly web application CandiSNP

**DOI:** 10.1186/s13007-014-0041-7

**Published:** 2014-12-30

**Authors:** Graham J Etherington, Jacqueline Monaghan, Cyril Zipfel, Dan MacLean

**Affiliations:** The Sainsbury Laboratory, Norwich Research Park, Norwich, NR4 7UH UK; The Genome Analysis Centre, Norwich Research Park, Norwich, NR4 7UH UK

**Keywords:** High-throughput sequencing, Single nucleotide polymorphisms, Forward-genetics, Mapping, Web application

## Abstract

**Background:**

Analysis of mutants isolated from forward-genetic screens has revealed key components of several plant signalling pathways. Mapping mutations by position, either using classical methods or whole genome high-throughput sequencing (HTS), largely relies on the analysis of genome-wide polymorphisms in F_2_ recombinant populations. Combining bulk segregant analysis with HTS has accelerated the identification of causative mutations and has been widely adopted in many research programmes. A major advantage of HTS is the ability to perform bulk segregant analysis after back-crossing to the parental line rather than out-crossing to a polymorphic ecotype, which reduces genetic complexity and avoids issues with phenotype penetrance in different ecotypes. Plotting the positions of homozygous polymorphisms in a mutant genome identifies areas of low recombination and is an effective way to detect molecular linkage to a phenotype of interest.

**Results:**

We describe the use of single nucleotide polymorphism (SNP) density plots as a mapping strategy to identify and refine chromosomal positions of causative mutations from screened plant populations. We developed a web application called CandiSNP that generates density plots from user-provided SNP data obtained from HTS. Candidate causative mutations, defined as SNPs causing non-synonymous changes in annotated coding regions are highlighted on the plots and listed in a table. We use data generated from a recent mutant screen in the model plant *Arabidopsis thaliana* as proof-of-concept for the validity of our tool.

**Conclusions:**

CandiSNP is a user-friendly application that will aid in novel discoveries from forward-genetic mutant screens. It is particularly useful for analysing HTS data from bulked back-crossed mutants, which contain fewer polymorphisms than data generated from out-crosses. The web-application is freely available online at http://candisnp.tsl.ac.uk.

**Electronic supplementary material:**

The online version of this article (doi:10.1186/s13007-014-0041-7) contains supplementary material, which is available to authorized users.

## Background

Carefully designed forward-genetic screens have been an integral part of research programs for decades and remain an important tool for resolving biological pathways. Many proteins contributing to plant immune signalling have been discovered through such screens. As one example, the receptor kinase FLAGELLIN SENSING 2 (FLS2) was identified from a mutagenized *Arabidopsis thaliana* (hereafter, Arabidopsis) population as the receptor for bacterial flagellin [1]. The discovery of FLS2 and other surface-localized immune receptors that detect conserved molecular features of microbes (known as pathogen-associated molecular patterns; PAMPs) revolutionized our understanding of plant immunity [2], and reinforces the importance of genetic screens in modern research.

Genetic screens in all systems are based on similar principles. Individuals containing a phenotype of interest are first isolated from a mutagenized or naturally polymorphic population. Marker-assisted linkage analysis is then performed to identify the genomic region containing the underlying mutation(s). Finally, mutations are identified by sequence analysis and the causative mutation is usually confirmed by complementation with a non-mutated (wild-type) copy of the gene.

The most commonly used mutagenesis strategies in Arabidopsis include the induction of guanine-to-adenine substitutions using ethylmethane sulfonate (EMS) or the insertion of transfer-DNA using *Agrobacterium tumefaciens*-mediated transformation [3]. The number of mutations identified in a mapped region depends primarily on the mutagenesis. Increasing the strength of the mutagen will likely result in the recovery of more mutants containing the phenotype of interest, however this also results in more mutations in each mutant genome and can complicate correct gene identification. Mapping mutations by position classically involves out-crossing to a polymorphic ecotype and linking the phenotype of recombinant F_2_ individuals to molecular markers with known genomic positions, such as insertion/deletions (indels) or single nucleotide polymorphisms (SNPs). Rather than genotyping individual recombinants exhibiting the scored phenotype, linkage analysis can be performed on bulked recombinants. This process, referred to as bulk segregant analysis (BSA) [4], eases genetic analysis and is particularly effective when a large number of molecular markers are available. Although robust, these classical methods are time-consuming and labour-intensive, commonly taking more than a year (in the case of Arabidopsis) to correctly identify the causative mutation. Positional cloning can be particularly tedious in suppressor or modifier screens, where multiple loci are segregating in the mapping population. Correct identification of causative mutations depends greatly on the strength of the mutagenesis, the complexity of the cross, the penetrance of the phenotype, and the availability of molecular markers.

Recent advances in high-throughput sequencing (HTS) technologies have allowed for rapid identification of causative mutations and have been widely adopted in many fields [5]. HTS approaches have many advantages over classical mapping strategies, including a potential reduction in time and personnel needed to identify a causative mutation. Combining HTS with BSA has proven to be particularly useful [5]. Several expert operator methods utilizing HTS of bulked segregants have been described to assist plant genetic screens, including SHOREmap [6,7], Next-Generation Mapping (NGM) [8], MutMap [9-11], and others [5]. However, each of these has restrictions in their functioning that make them limited to certain types of crosses or are difficult to use for non-experts including bench-trained biologists.

The two major tools used by plant researchers start with a classical mapping approach, requiring data generated from out-crosses. SHOREmap [6,7] uses a statistic that explicitly calculates the relative abundance of alleles identified in bulked out-crossed F_2_ populations and therefore relies on *a priori* knowledge of polymorphic allele positions in both the parental and out-crossed ecotypes. As a result, this powerful tool cannot be used when such crosses are not performed (for example, in back-crossed populations) or when genetic marker resources are not yet available. Comparatively, the marker-independent, web-based method NGM [8], does not rely on previous knowledge of ecotype-specific polymorphisms, but rather uses the ratio of the expected allele frequency of the causative mutation in bulked out-cross F_2_ segregants relative to the background allele frequency of unrelated mutations. This two-step process relies first on identifying a coarse mapping interval of relative SNP paucity in which the mutation should lie; this region is usually in the order of megabases in length [8]. To further reduce the width of the coarse interval and ease identification of the causative mutation, the SNP frequency in different bands of SNP allele frequencies (overlapping groupings of similar allele frequencies; *e.g*., 0.5-0.6, 0.51-0.61, etc.) are compared. The point that maximises the ratio between bands representing homozygous alleles and heterozygous alleles at 50% frequency is expected to be at or around the causative mutation. The MutMap [10,11] and CloudMap [12] systems avoid the need for coarse mapping as an initial step but do not exist as user-friendly tools such that application and optimisation of parameters requires extensive expertise in a command-line computing environment.

We recently conducted a forward-genetic modifier screen in the immune-deficient *bak1*-*5* background to identify novel components involved in plant immune signalling [13]. BRASSINOSTEROID INSENSITIVE 1-ASSOCIATED KINASE 1 (BAK1) is a multi-functional co-receptor that interacts with and phosphorylates several surface-localized immune receptors including FLS2 [14-19]. Accordingly, loss-of-function *bak1* alleles are strongly impaired in signalling triggered by several PAMPs [15,17-19]. We mutagenized *bak1*-*5* seeds (in the Columbia-0 (Col-0) ecotype) with EMS and screened the M_2_ generation for *modifier of bak1*-*5* (*mob*) mutants that restored immune signalling. To uncover the causal *mob* mutation(s), we back-crossed *bak1*-*5 mob* mutants to the parental line (*bak1*-*5*) and Illumina sequenced bulked F_2_*mob* segregants. Importantly, as the parent was itself generated through EMS-mutagenesis of a transgenic Arabidopsis line [15,20,21], we additionally sequenced *bak1*-*5*, which had been back-crossed for three generations prior to mutagenesis, as a reference.

We chose this approach over out-crossing to ease phenotyping and segregation analysis. First, selection of the *mob* mutant phenotype required scoring a quantitative response dependent on immune receptor activity, which varies in different ecotypes. For example, *FLS2* in the Wassilewskija-0 (Ws-0) ecotype contains a deletion mutation resulting in a truncated and non-functional FLS2 receptor [22], while *FLS2* in the Landsberg *erecta*-0 (L*er*-0) ecotype contains polymorphisms that cause FLS2 to bind flagellin about three times stronger than FLS2 in Col-0 [23]. Second, selection of the *mob* mutant phenotype was dependent on *bak1*-*5*, which would not be present in a polymorphic ecotype such as L*er*-0 and would thus need to be genotyped prior to phenotype scoring. Similar considerations would likely arise in any screen involving second-site modifier or suppressor mutations.

While a back-cross simplifies genetic analysis, bulk segregant sequence analysis is complicated by far fewer segregating SNPs (1 SNP every 65,000 bp) compared to out-crosses (1 SNP every 900 bp) (Additional file 1). Although few in number, we found that simply plotting the position of SNPs with close-to-homozygous alternate allele frequencies along the chromosomes was a convenient and easy way of performing bulk segregant linkage analysis from a back-crossed population. We developed this method into a user-friendly web-based application, called CandiSNP, which generates density plots from SNP data obtained from HTS. We demonstrate the utility of CandiSNP by analysing sequence data generated from two allelic *mob* mutants, *bak1*-*5 mob1* and *bak1*-*5 mob2*, which are caused by mutations in the gene encoding the calcium-dependent protein kinase CPK28 [13].

### Implementation

#### CandiSNP is part of a straightforward and flexible workflow

**Figure 1:**
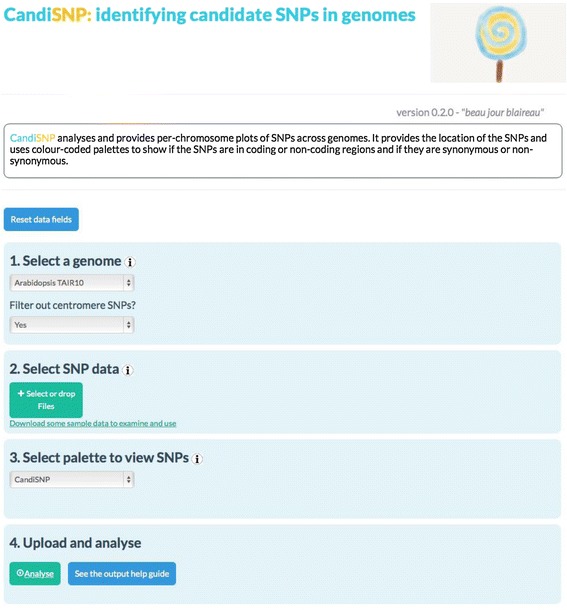
**Screen-shot of the CandiSNP web application.** CandiSNP is openly accessible online at http://candisnp.tsl.ac.uk. The application is laid out so users can make their way through the application in numbered steps. Users choose which genome they would like to use for comparison (the program currently supports Arabidopsis, rice, tomato, grape, maize and soybean genomes). The option of filtering SNPs concentrated around centromeres is also provided. Users then upload their SNP data file, indicate their preferred allele frequency cut-off, and choose from a number of different palettes for SNP visualization.

**Figure 2:**
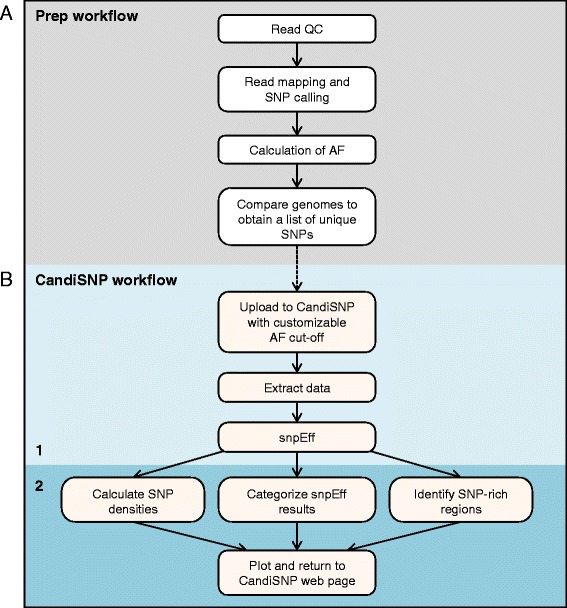
**Bioinformatics pipeline for sequence analysis.** Pipeline indicating the preparatory steps required by the user **(A)** prior to running the CandiSNP web application **(B)**.

To provide a publicly available easy-to-use tool for mapping mutations, we developed the CandiSNP web application (Figure 1). Prior to using CandiSNP, users must identify SNP positions. Typically this would be done in a workflow that maps quality-controlled (QC) reads to the appropriate reference genome and provides SNP position data as input (Figure 2A). The data must be provided in a simple comma-delimited format and must have the following column headers: ‘Chr’, ‘Pos’, ‘Ref’, ‘Alt’ and ‘Allele_Freq’ (meaning: Chromosome, Position, Reference base, Alternate base, and Allele Frequency, respectively). To create CandiSNP input files, we suggest using the pileups_to_snps.rb Ruby script (Additional file 2 and https://github.com/danmaclean/candisnp/blob/master/pileup_to_snps.rb). By using this generic and flexible input format, our system allows the user to take advantage of existing pipelines and data, including files generated by external service providers or even datasets originating from other technologies.

CandiSNP analysis is a two-step process (Figure 2B). CandiSNP first uses snpEff [24] to categorise SNPs according to their position in genomic features. The SNP predictions are categorised as: (1) Causing a change in an intergenic (non-annotated) region, (2) Causing a synonymous change in an annotated protein-coding region, or (3) Causing a non-synonymous change in an annotated protein-coding region. CandiSNP then creates a chromosome map visualizing the position of all SNPs meeting a user-selected (and customizable) alternate allele frequency (AF) threshold, and renders this information by colouring SNPs according to category. SNPs in category (3) are represented in a colour that highlights their priority as putative causative SNPs. The density and distribution of SNPs is also visualized as a line graph below each chromosome. The user is provided with a downloadable list of genomic effects for all inputted SNPs and is provided with a Scalable Vector Graphic figure of publication quality that can be easily exported.

Currently, CandiSNP supports several plant genomes including *Arabidopsis thaliana* Col-0 TAIR9 and TAIR10 [25], *Oryzae sativa* v7 [26], *Solanum lycopersicum* v2.40 [27], *Glycine max* 1.09v8 [28], *Vitus vinefera* v1 [29] and *Zea mays* B73 v5b [30].

### Design and availability

CandiSNP is available in multiple formats for users with diverse security and confidentiality needs and differing access to computational infrastructure. Primarily, CandiSNP is provided as a web application, available at http://candisnp.tsl.ac.uk. The web application takes text files as input. Instructions are provided on-screen to assist users new to the tool. The web application requires no registration and does not collect user information. For laboratories with bioinformatics support wishing to use an internal and private version of the web application, we provide a package and source code in Perl/HTML/Javascript for free download and use under the GNU GPL3 Licence, from the dedicated code hosting website GitHub at https://github.com/danmaclean/candisnp. For those wishing to run the CandiSNP process on a command line as part of bioinformatics pipelines, a Perl module is also available as part of the source code.

## Results and discussion

### Case study

#### *Bulk segregant analysis of two* mob *mutants using Illumina sequencing identifies thousands of polymorphisms*

**Figure 3:**
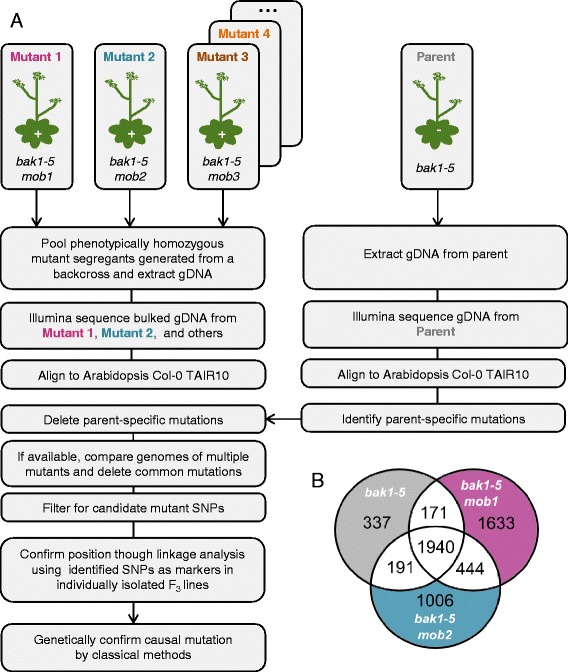
**Pipeline for bulking segregants and identification of unique SNPs. (A)** The recessive *bak1*-*5 mob1* and *bak1*-*5 mob2* mutants were back-crossed to the parent *bak1*-*5*, allowed to self-fertilize in the F_1_, and phenotypically scored in the F_2_ for the *mob* phenotype. Positive segregants were bulk harvested and genomic DNA was prepared and sequenced using the Illumina HiSeq platform. For comparison, the *bak1*-*5* genome was also sequenced. A similar genetics pipeline could be employed for dominant mutants, but material would need to be bulked from segregants that were phenotypically verified as homozygous in the F_3_ generation. **(B)** A three-way comparison between the *bak1*-*5*, *bak1*-*5 mob1*, and *bak1*-*5 mob2* genomes identified the total number of unique SNPs in each genome.

As a case study for CandiSNP we examined HTS data obtained for two allelic recessive mutants, *bak1*-*5 mob1* and *bak1*-*5 mob2*, that were isolated from the *modifier of bak1*-*5* (*mob*) screen [13]. Both *bak1*-*5 mob1* and *bak1*-*5 mob2* were back-crossed with *bak1*-*5* and the F_2_ populations were screened for the *mob* phenotype (Figure 3A). F_2_ segregants that displayed the *mob* phenotype were bulked and genomic DNA was isolated. The *bak1*-*5* parental genome was prepared by harvesting individuals from a homozygous back-crossed line. DNA samples were sent to the Beijing Genomics Institute (BGI, Hong Kong) for library construction and 90 bp paired-end sequencing on the Illumina HiSeq platform.

With the aid of FASTQC v 0.10.1 [31] in the Galaxy platform [32-34], all reads were quality controlled (QC) so that reads that contained undefined nucleotides, were not 90 bp long, or were full-length homopolymer runs were removed. Reads containing nucleotides with a Sanger-scaled PHRED quality score of less than 10 at the 3′ end were trimmed to this minimum using Sickle version 1.21.0 [35]. The QC pipeline is available as a Galaxy workflow at http://dx.doi.org/10.6084/m9.figshare.1248898.

QC reads were then mapped to the TAIR10 genome [25] using the BWA v 0.6.1 aligner [36]. For the *bak1*-*5* genome, we aligned 47.9 million 90 bp paired-end reads, with a mean insert size of 467 bp, to the TAIR10 Arabidopsis reference sequence (98.5% of reads aligned). We similarly aligned 47.2 million 90 bp paired-end reads, with a mean insert size of 452 bp, to TAIR10 for the *bak1*-*5 mob2* genome (99.6% of reads aligned). Average alignment depth over the nuclear chromosomes was 36 for *bak1*-*5* and 59.3 for *bak1*-*5 mob2*. Details regarding the *bak1*-*5 mob1* genome sequence have been previously described [13].

After alignment, SNPs were identified and allele frequencies were calculated using SAMtools v 0.1.8 [37]. Reads with mapping quality scores less than 20 and individual bases with sequence quality less than 20 were discarded. Genome positions where the reference base was unknown were excluded. Positions were considered SNPs if they had a minimum read coverage of 6 and a maximum of 250. The alignment and SNP calling workflow is available at http://dx.doi.org/10.6084/m9.figshare.1171109. In total, *bak1*-*5* contained 2,639 SNPs compared to Col-0, while *bak1*-*5 mob1* and *bak1*-*5 mob2* contained 4,188 and 3,581 SNPs, respectively (Table 1).

#### Filtering of non-unique SNPs in the mutants reduces complexity

To reduce the complexity of the *bak1*-*5 mob1* and *bak1*-*5 mob2* datasets, we compared SNP calls from the different genome sequences and removed SNPs that either *mob* had in common with each-other or *bak1*-*5*. Comparing the *bak1*-*5 mob* genomes to the parental *bak1*-*5* genome identified 2,111 and 2,132 SNPs shared between *bak1*-*5* and either *bak1*-*5 mob1* or *bak1*-*5 mob2*, respectively (Figure 3B). There were an additional 444 SNPs that were shared between the *bak1*-*5 mob1* and *bak1*-*5 mob2* genomes that were not identified in *bak1*-*5*. We reasoned that these shared SNPs were contributed by *bak1*-*5* but were not identified due to low sequence coverage in those areas. These analyses identified 2,746 polymorphisms that were shared between at least two of the genomes. Discarding these allowed us to identify over 1,000 SNPs that were uniquely present in *bak1*-*5 mob1* and *bak1*-*5 mob2* (Figure 3B). The general value of comparing multiple mutant sequences to remove shared SNPs has been previously demonstrated [12] and the general case discussed in Additional file 3. However, deleting all common SNPs in this way precludes identification of identical causative SNPs in different mutants, which, while extremely rare, is something to consider prior to performing such analysis.

#### CandiSNP enables easy visual assessment of SNP positions and finds genomic regions with low recombination linked to the phenotype of interest

Positional cloning is based on linking phenotypes to molecular markers with known genomic positions. If recessive, F_2_ recombinants that contain the phenotype of interest are homozygous for the unknown mutation. Identifying molecular markers that are invariably homozygous for the mutant type are therefore linked to the scored phenotype and thus the mutation. As we conducted a back-cross rather than an out-cross, the only molecular markers we could use were those resulting from the comparative analysis just described between the parental and mutant genomes.

**Figure 4:**
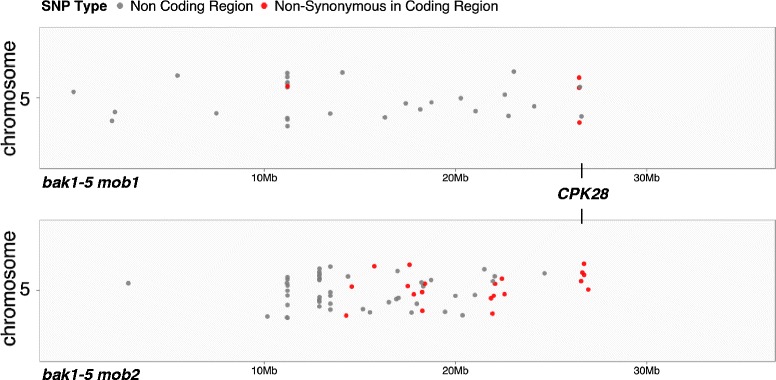
**Chromosome 5 SNP density plots for** ***bak1***-***5 mob1*** **and** ***bak1***-***5 mob2***. All SNPs with allele frequencies >75% are plotted in grey, while candidate causative SNPs (defined as those causing non-synonymous changes in gene-coding regions) are plotted in red. The position of *CPK28*/*At5g66210* is indicated.

To identify which of the over 1,000 unique SNPs in the mutant genomes are linked to the scored phenotype, it is necessary to determine which SNPs are homozygous or close-to-homozygous. Although bulking mutants increases the likelihood of identifying homozygous SNPs (in theory, with an allele frequency of 100%), some margin of error must be allowed to account for sequencing and phenotyping errors. CandiSNP facilitates the easy discovery of a useful frequency cut-off by allowing the user to iteratively refine the allele frequency and view a new plot concurrent with previous ones for comparison. As a further refinement of the CandiSNP web application, we included the option of removing SNPs concentrated around centromeres (in organisms where a centromere is defined in the genome assembly), as these are areas of low recombination frequency and tend to skew density analysis. After selecting an allele frequency threshold, CandiSNP plots the positions of retained SNPs as dots across the chromosomes and highlights SNPs of different classes according to a selected palette. The per-chromosome density and distribution of SNPs is rendered in a second plot to aid in cases with high numbers of SNPs. In our case study we chose 75% as an acceptable frequency cut-off, and used CandiSNP to identify 88 and 143 unique SNPs meeting this requirement in the *bak1*-*5 mob1* (Additional file 4) and *bak1*-*5 mob2* (Additional file 5) genomes, respectively (Table 1). CandiSNP further identified 9 and 31 candidate causative SNPs (those which cause non-synonymous changes in protein-coding regions) for *bak1*-*5 mob1* (Table 2; Additional file 4) and *bak1*-*5 mob2* (Table 3; Additional file 5). By choosing a palette to highlight candidate SNPs (shown in our case study as red dots) we observe putative map positions for both mutants at the bottom of chromosome 5 (Figure 4).

**Table 1:** **Identification of unique and candidate SNPs in the parental and mutant genomes**

	***bak1*** **-** ***5***	***bak1*** **-** ***5 mob1***	***bak1*** **-** ***5 mob2***
Total SNPs compared to Col-0 TAIR10	2639	4188	3581
Unique SNPs compared to the parent	2639	1633	1006
Unique SNPs, AF >75%	785	88	143
Unique SNPs, AF >75%, annotated coding	240	16	41
Unique SNPs, AF >75%, annotated coding, non-synon	168	9^a^	31^b^

To identify SNPs unique to each genome, the parental and mutant genomes were compared and filtered. Unique SNPs in *bak1*-*5* refer to those that are not found in the Col-0 TAIR10 genome. For the *bak1*-*5 mob1* and *bak1*-*5 mob2* datasets, SNPs shared between any of the three genomes (*bak1*-*5*, *bak1*-*5 mob1* and *bak1*-*5 mob2*; Figure 3B) were removed, resulting in SNPs uniquely found in each of those genomes. Filtering for SNPs with an allele frequency higher than 75% that cause non-synonymous (‘non-synon’) changes in annotated coding regions resulted in a list of candidate causative mutations.

^a^Candidate causative SNPs for *bak1*-*5 mob1* are listed in Table 2.

^b^Candidate causative SNPs for *bak1*-*5 mob2* are listed in Table 3.

**Table 2:** **Candidate causative SNPs in** ***bak1*** **-** ***5 mob1***

**Chr**	**Position**	**Ref/Alt**	**AF (%)**	**AGI**	**Gene ID**	**AA change**	**Sanger F**_**3**_
1	11892068	C/T	100	At1g32830	Transposable element	n.a.	Absent
1	11892070	G/T	100	At1g32830	Transposable element	n.a.	Hom^a^
1	11892252	T/G	100	At1g32830	Transposable element	n.a.	Hom^a^
1	16516501	T/C	83.3	At1g43745	Transposable element	n.a.	Absent
1	16525522	T/C	77.8	At1g43755	Transposable element	n.a.	Hom^a^
1	24243231	G/A	80.9	At1g65270	Unknown protein	G > S	Hom
5	26457834	G/A	85.0	At5g66210	CPK28	A > V	Hom^b^
5	26458077	G/A	78.5	At5g66210	CPK28	S > L	Hom^b^
5	26474069	G/A	76.5	At5g66270	Zn-finger family protein	P > L	Hom

Unique SNPs in annotated coding regions with allele frequencies (AF) over 75% identified by CandiSNP for *bak1*-*5 mob1*, listing the Chromosome number (Chr), position, reference base (Ref), sequenced alternate base (Alt), locus number (AGI), gene identification (Gene ID), amino acid change (AA change; ‘n.a.’ is not applicable). All SNPs were confirmed in at least three independent back-crossed lines (F_3_ generation) by Sanger sequencing compared to *bak1*-*5*. SNPs that were homozygous (Hom) or not present (Absent) are listed.

^a^These SNPs were also identified in *bak1*-*5* by Sanger sequencing (however, not by Illumina sequencing) and are therefore not unique to *bak1*-*5 mob1*.

^b^These SNPs are the causative mutations for *bak1*-*5 mob1* [13].

**Table 3:** **Candidate causative SNPs in** ***bak1*** **-** ***5 mob2***

**Chr**	**Position**	**Ref/ Alt**	**AF (%)**	**AGI**	**Gene ID**	**AA change**	**Sanger F**_**3**_
1	7446564	C/T	76.2	At1g21270	WAK2	P > L	Seg
1	11892984	C/T	100	At1g32830	Transposable element	n.a.	Hom^a,b^
1	16513961	T/G	77.1	At1g43740	Transposable element	n.a.	Hom^a,b^
1	17757465	C/T	76.0	At1g48090	Calcium-dependent lipid-binding protein	D > N	Seg
1	18192647	C/T	76.0	At1g49190	ARR19	R > W	Seg
1	22178447	C/T	82.6	At1g60140	TPS10	D > N	Hom
2	2568811	C/T	83.3	At2g06470	Transposable element	n.a.	Hom^a^
2	5277241	T/G	100	At2g12850	Transposable element	n.a.	Not tested
4	2362567	C/A	100	At4g04655	Transposable element	n.a.	Seg^a^
5	5569896	G/A	94.1	At5g16930	AAA-type ATPase family protein	W > stop	Seg
5	14285189	G/A	81.3	At5g36260	Eukaryotic aspartyl protease family protein	S > L	Absent
5	14579245	G/A	75.0	At5g36935	Transposable element	n.a.	Not tested
5	15751875	G/A	77.2	At5g39350	Tetratricopeptide repeat-like superfamily protein	R > K	Seg
5	17503318	G/A	82.7	At5g43560	TRAF-like superfamily protein	E > K	Seg
5	17597830	G/A	80.0	At5g43800	Transposable element	n.a.	Not tested
5	17820568	G/A	93.3	At5g44240	ALA2	A > T	Seg
5	18251689	G/A	83.3	At5g45140	Nuclear RNAP2	P > S	Seg
5	18261108	G/A	75.0	At5g45150	RTL3	D > N	Seg
5	18399206	G/A	80.9	At5g45400	RPA70C	V > M	Seg
5	21859555	G/A	90.0	At5g53840	F-box/RNI-like/FBD-like domains-containing protein	S > F	Seg
5	21939106	G/A	80.9	At5g54062	Unknown protein	E > K	Seg
5	22002355	G/A	88.9	At5g54203	Transposable element	n.a.	Absent
5	22066915	G/A	79.3	At5g54340	C2H2 and C2HC zinc-finger superfamily protein	V > I	Seg
5	22430866	G/A	90.0	At5g55310	TOP1β	A > V	Seg
5	22565056	G/A	76.5	At5g55750	Hydroxyproline-rich glycoprotein family protein	P > S	Seg
5	26458017	C/T	93.1	At5g66210	CPK28	W > stop	Hom^c^
5	26560691	C/T	95.8	At5g66550	Maf-like protein	G > R	Hom
5	26626055	C/T	78.9	At5g66690	UGT72E2	P > S	Hom
5	26710709	C/T	75.0	At5g66880	SNRK2.3	P > S	Hom
5	26716839	C/T	80.9	At5g66900	CC-NB-LRR family protein	D > N	Hom
5	26935248	C/T	75.0	At5g67500	VDAC2	T > I	Hom

Unique SNPs in annotated coding regions with allele frequencies (AF) over 75% identified by CandiSNP for *bak1*-*5 mob2*, listing the Chromosome number (Chr), position, reference base (Ref), sequenced alternate base (Alt), locus number (AGI), gene identification (Gene ID), amino acid change (AA change; ‘n.a.’ is not applicable). All SNPs were confirmed in at least three independent back-crossed lines (F_3_ generation) by Sanger sequencing compared to *bak1*-*5*. SNPs that were homozygous (Hom), segregating (Seg), not identified (Absent), or not tested are listed.

^a^These SNPs were also identified in *bak1*-*5* by Sanger sequencing (however, not by Illumina sequencing) and are therefore not unique to *bak1*-*5 mob2*.

^b^These are single base deletion mutations.

^c^This SNP is the causative mutation for *bak1*-*5 mob2* [13].

Therefore, CandiSNP visualizes the location of SNPs linked to a phenotype of interest. Moreover, CandiSNP provides annotations describing the genomic feature in which each SNP is located. This function provides useful information for biologists who can make conceptual links between biological knowledge and molecular function of the genomic feature and refine candidate lists further. On its own this annotation function provides a fast and easy way of finding the effect of any mutation in the supported genomes, enhancing the usefulness of CandiSNP beyond that of mutant mapping.

#### Fine mapping and confirmation of causative SNPs

We confirmed the presence of candidate causative SNPs by Sanger sequencing polymerase chain reaction (PCR)-generated amplicons containing the predicted mutations prepared from individual back-crossed F_3_*bak1*-*5 mob1* and *bak1*-*5 mob2* plants compared to the *bak1*-*5* parent (Tables 2 and 3). Using these SNPs as molecular markers allowed us to further map the mutations by position and narrowed the list of candidate causative SNPs down to 3 in *bak1*-*5 mob1* and 6 in *bak1*-*5 mob2*. Primers used for this analysis are available in Additional file 6. Alternative methods for such analysis could include other allele-specific genotyping methods such as designing cleaved amplified polymorphic sequence (CAPS) markers [38] or conducting high-resolution melting (HRM) analysis [39] on PCR amplicons. We previously reported genetic confirmation that the polymorphic *CPK28* alleles contained within these lists of candidate SNPs were causative of the *bak1*-*5 mob1* and *bak1*-*5 mob2* mutant phenotypes [13]. Our analysis was simplified by knowledge of allelism between the two mutants, which clearly indicated *CPK28* as the causative locus. In the absence of such knowledge, marker-assisted genotyping of additional homozygous F_3_ lines could further reduce the number of candidate mutations and ease genetic confirmation.

#### CandiSNP performance

As CandiSNP is a predictive classification method for determining whether a given SNP is a causative mutation, it is important that we estimate the accuracy of the classifications made. One useful approach for assessing the overall accuracy of the analysis, rather than each individual prediction, *post hoc*, is to construct a receiver operating characteristic (ROC) curve [40]. In such an analysis, a set of independently verified ‘true positive’ results are compiled and the ability of the classifier to recall these at different parameters is plotted. To assess CandiSNP we used the *bak1*-*5 mob1* and *bak1*-*5 mob2* verified causative SNPs and varied the allele frequency parameter to carry out a standard ROC analysis [40] (Additional file 7). True positives were defined as verified causative SNPs and false positives were defined as any non-causative SNP identified by CandiSNP regardless of location and category. False negatives were defined as causative SNPs not included in that threshold and true negatives as any position in the genome where a SNP was not identified (*i.e*., genome size – false positives). Sensitivity was calculated as the number of true positives divided by the total of true positives and false negatives. Specificity was calculated as the number of true negatives divided by the number of false positives and true negatives. Sensitivity assesses the ability of CandiSNP to recall the verified SNPs whilst specificity assesses the ability of CandiSNP to exclude non-causative SNPs.

For our test case, the sensitivity of CandiSNP drops completely for allele frequencies over 75% (Additional file 7), indicating that accounting for phenotype penetrance and sequencing errors is an important factor in the pipeline. Further, rather than reducing the number of errors, setting an overly stringent allele frequency causes the pipeline to fail by screening out real candidates. Specificity remains high across all possible allele frequencies, mostly due to the masking effect of a very high true negative count. The closely related false positive score shows a decrease to less than 25% of the candidate SNP list after an allele frequency of 62%. While the absolute optimum for our data is at 75% (*i.e*., the maximisation of sensitivity and minimisation of false positives), taken as a whole the ROC analysis indicates that allele frequencies of 60% to 75% represent a likely ‘best trade-off’ window for CandiSNP analysis.

## Conclusions

Genetic screens have revealed important regulators of signal transduction pathways and remain an important tool in modern research. Although greatly accelerated with the advent of HTS technologies, correct identification of causative mutation(s) remains a bottleneck in forward-genetics. To increase the repertoire of programs available to plant geneticists, we developed the CandiSNP web application, which is particularly useful for datasets containing few SNPs. In our test case, CandiSNP successfully identified causative SNPs in two recessive mutants after bulking phenotypically homozygous F_2_ segregants generated from a back-cross. We propose that CandiSNP could additionally be used to identify causative SNPs in dominant mutants as long as they are verified to be phenotypically homozygous in the F_3_ prior to bulk segregant analysis. By plotting homozygous and close-to-homozygous SNPs identified from HTS along the chromosome arms, the program visualizes areas of linkage and easily narrows down candidate mutation positions. CandiSNP is both fast and accurate, producing high-quality editable graphics in a matter of minutes. CandiSNP is a user-friendly web application that will facilitate gene discovery in plant genetic screens.

### Availability requirements

**Project name**: CandiSNP.

**Project home page**: http://candisnp.tsl.ac.uk

(Source code is available under the GPLv3 open-source license at https://github.com/danmaclean/candisnp).

**Operating system(s)**: All systems capable of running a modern web-browser.

**Other requirements**: Internet connection.

**License**: GPL3 (http://www.gnu.org/licenses/licenses.html).

**Any restrictions**: None.

## Additional files

Additional file 1:
**Calculation of expected SNP frequencies in out- and back-crosses.** SNP frequencies are calculated from different types of crosses using available data. Additional file 2:
**SNP calling script.** To create our CandiSNP input files we used the pileups_to_snps.rb Ruby script which relies on the bio-samtools and bio-gngm Ruby Gems. The input data is the FASTA reference sequence (TAIR10 genome in this case) and the SAM/BAM alignment file for each dataset. The output is a comma-delimited file of SNPs formatted for input to the CandiSNP application. The source code for this script is available at https://github.com/danmaclean/candisnp/blob/master/pileup_to_snps.rb. Additional file 3:
**Simplifying SNP analysis by comparing multiple mutant genomes.** Comparing multiple mutant genomes identifies unique SNPs in each mutant. Additional file 4:
**Genome-wide SNP density plots for** ***bak1*** **-** ***5 mob1.*** Using the ‘CandiSNP palette’, all SNPs with allele frequencies >75% are plotted in grey, while candidate causative SNPs (defined as those causing non-synonymous changes in gene-coding regions) are plotted in red. These plots are also available at http://dx.doi.org/10.6084/m9.figshare.1250028. Additional file 5:
**Genome-wide SNP density plots for** ***bak1*** **-** ***5 mob2.*** Using the ‘CandiSNP palette’, all SNPs with allele frequencies >75% are plotted in grey, while candidate causative SNPs (defined as those causing non-synonymous changes in gene-coding regions) are plotted in red. These plots are also available at http://dx.doi.org/10.6084/m9.figshare.1250027. Additional file 6:
**Primers used in this study.** Genomic regions containing candidate SNPs in *bak1*-*5 mob1* and *bak1*-*5 mob2* were amplified by PCR using these primers and Sanger-sequenced alongside *bak1*-*5* as a control. Additional file 7:
**Receiver operating characteristic curve plot.** Receiver operating characteristic curve plot, demonstrating the optimal parameters for use in CandiSNP on *bak1*-*5 mob1*. The proportion of false positives (non-causative SNPs identified by CandiSNP regardless of location and category) is also plotted as a comparison to the standard sensitivity and specificity [40]. The overlaid dashed line represents the optimal point that maximises the ability of CandiSNP to find real causative SNPs (sensitivity) while minimising the inclusion of false positives.

## Abbreviations

AF: Allele frequency
Alt: Alternative sequence
BAK1: BRASSINOSTEROID INSENSITIVE 1-ASSOCIATED KINASE 1
Bp: base pair
BWA: Burrows-Wheeler aligner
CAPS: Cleaved amplified polymorphic sequence
CandiSNP: Candidate SNP
Chr: Chromosome
CPK28: CALCIUM-DEPENDENT PROTEIN KINASE 28
Col-0: Columbia-0
EMS: Ethylmethane sulfonate
F: Filial generation
FLS2: FLAGELLIN SENSING 2
HRM: High-resolution melting
Indel: Insertion/deletion
L*er*-0: Landsberg *erecta*-0
M: Mutant generation
*Mob*: *modifier of bak1*-*5*
NGM: Next-generation mapping
HTS: High-throughput sequencing
PCR: Polymerase chain reaction
Pos: Position
SAM: Sequence alignment/map
SNP: Single nucleotide polymorphism
TAIR: The Arabidopsis Information Resource
QC: Quality control
Ws-0: Wassilewskija-0

## References

[1] Gomez-Gomez L, Boller T (2000). FLS2: an LRR receptor-like kinase involved in the perception of the bacterial elicitor flagellin in Arabidopsis. Mol Cell.

[2] Boller T, Felix G (2009). A renaissance of elicitors: perception of microbe-associated molecular patterns and danger signals by pattern-recognition receptors. Annu Rev Plant Biol.

[3] Page DR, Grossniklaus U (2002). The art and design of genetic screens: Arabidopsis thaliana. Nat Rev Genet.

[4] Michelmore RW, Paran I, Kesseli RV (1991). Identification of markers linked to disease-resistance genes by bulked segregant analysis: a rapid method to detect markers in specific genomic regions by using segregating populations. Proc Natl Acad Sci U S A.

[5] Schneeberger K (2014). Using next-generation sequencing to isolate mutant genes from forward genetic screens. Nat Rev Genet.

[6] Schneeberger K, Ossowski S, Lanz C, Juul T, Petersen AH, Nielsen KL, Jorgensen JE, Weigel D, Andersen SU (2009). SHOREmap: simultaneous mapping and mutation identification by deep sequencing. Nat Methods.

[7] Hartwig B, James GV, Konrad K, Schneeberger K, Turck F (2012). Fast isogenic mapping-by-sequencing of ethyl methanesulfonate-induced mutant bulks. Plant Physiol.

[8] Austin RS, Vidaurre D, Stamatiou G, Breit R, Provart NJ, Bonetta D, Zhang J, Fung P, Gong Y, Wang PW, McCourt P, Guttman DS (2011). Next-generation mapping of Arabidopsis genes. Plant J.

[9] Takagi H, Uemura A, Yaegashi H, Tamiru M, Abe A, Mitsuoka C, Utsushi H, Natsume S, Kanzaki H, Matsumura H, Saitoh H, Yoshida K, Cano LM, Kamoun S, Terauchi R (2013). MutMap-Gap: whole-genome resequencing of mutant F2 progeny bulk combined with de novo assembly of gap regions identifies the rice blast resistance gene Pii. New Phytol.

[10] Fekih R, Takagi H, Tamiru M, Abe A, Natsume S, Yaegashi H, Sharma S, Kanzaki H, Matsumura H, Saitoh H, Mitsuoka C, Utsushi H, Uemura A, Kanzaki E, Kosugi S, Yoshida K, Cano L, Kamoun S, Terauchi R (2013). MutMap+: genetic mapping and mutant identification without crossing in rice. PLoS One.

[11] Abe A, Kosugi S, Yoshida K, Natsume S, Takagi H, Kanzaki H, Matsumura H, Mitsuoka C, Tamiru M, Innan H, Cano L, Kamoun S, Terauchi R (2012). Genome sequencing reveals agronomically important loci in rice using MutMap. Nat Biotechnol.

[12] Minevich G, Park DS, Blankenberg D, Poole RJ, Hobert O (2012). CloudMap: a cloud-based pipeline for analysis of mutant genome sequences. Genetics.

[13] Monaghan J, Matschi S, Shorinola O, Rovenich H, Matei A, Segonzac C, Gro-Malinovsky F, Rathjen J, MacLean D, Romeis T, Zipfel C (2014). The calcium dependent protein kinase CPK28 buffers plant immunity and regulates BIK1 turnover. Cell Host Microbe.

[14] Sun Y, Li L, Macho AP, Han Z, Hu Z, Zipfel C, Zhou JM, Chai J (2013). Structural basis for flg22-induced activation of the Arabidopsis FLS2-BAK1 immune complex. Science.

[15] Schwessinger B, Roux M, Kadota Y, Ntoukakis V, Sklenar J, Jones A, Zipfel C (2011). Phosphorylation-dependent differential regulation of plant growth, cell death, and innate immunity by the regulatory receptor-like kinase BAK1. PLoS Genet.

[16] Schulze B, Mentzel T, Jehle AK, Mueller K, Beeler S, Boller T, Felix G, Chinchilla D (2010). Rapid heteromerization and phosphorylation of ligand-activated plant transmembrane receptors and their associated kinase BAK1. J Biol Chem.

[17] Roux M, Schwessinger B, Albrecht C, Chinchilla D, Jones A, Holton N, Malinovsky FG, Tor M, de Vries S, Zipfel C (2011). The arabidopsis leucine-rich repeat receptor-like kinases BAK1/SERK3 and BKK1/SERK4 are required for innate immunity to Hemibiotrophic and Biotrophic pathogens. Plant Cell.

[18] Heese A, Hann DR, Gimenez-Ibanez S, Jones AM, He K, Li J, Schroeder JI, Peck SC, Rathjen JP (2007). The receptor-like kinase SERK3/BAK1 is a central regulator of innate immunity in plants. Proc Natl Acad Sci U S A.

[19] Chinchilla D, Zipfel C, Robatzek S, Kemmerling B, Nurnberger T, Jones JD, Felix G, Boller T (2007). A flagellin-induced complex of the receptor FLS2 and BAK1 initiates plant defence. Nature.

[20] Nekrasov V, Li J, Batoux M, Roux M, Chu ZH, Lacombe S, Rougon A, Bittel P, Kiss-Papp M, Chinchilla D, van Esse HP, Jorde L, Schwessinger B, Nicaise V, Thomma BP, Molina A, Jones JD, Zipfel C (2009). Control of the pattern-recognition receptor EFR by an ER protein complex in plant immunity. EMBO J.

[21] Tedman-Jones JD, Lei R, Jay F, Fabro G, Li X, Reiter WD, Brearley C, Jones JD (2008). Characterization of Arabidopsis mur3 mutations that result in constitutive activation of defence in petioles, but not leaves. Plant J.

[22] Zipfel C, Robatzek S, Navarro L, Oakeley EJ, Jones JD, Felix G, Boller T (2004). Bacterial disease resistance in Arabidopsis through flagellin perception. Nature.

[23] Vetter MM, Kronholm I, He F, Haweker H, Reymond M, Bergelson J, Robatzek S, de Meaux J (2012). Flagellin perception varies quantitatively in Arabidopsis thaliana and its relatives. Mol Biol Evol.

[24] Cingolani P, Platts A, le Wang L, Coon M, Nguyen T, Wang L, Land SJ, Lu X, Ruden DM (2012). A program for annotating and predicting the effects of single nucleotide polymorphisms, SnpEff: SNPs in the genome of Drosophila melanogaster strain w1118; iso-2; iso-3. Fly (Austin).

[25] Huala E, Dickerman AW, Garcia-Hernandez M, Weems D, Reiser L, LaFond F, Hanley D, Kiphart D, Zhuang M, Huang W, Mueller LA, Bhattacharyya D, Bhaya D, Sobral BW, Beavis W, Meinke DW, Town CD, Somerville C, Rhee SY (2001). The Arabidopsis Information Resource (TAIR): a comprehensive database and web-based information retrieval, analysis, and visualization system for a model plant. Nucleic Acids Res.

[26] Project IRGS (2005). The map-based sequence of the rice genome. Nature.

[27] Consortium TTG (2012). The tomato genome sequence provides insights into fleshy fruit evolution. Nature.

[28] Schmutz J (2010). Genome sequence of the palaeopolyploid soybean. Nature.

[29] Velasco R, Zharkikh A, Troggio M, Cartwright DA, Cestaro A, Pruss D, Pindo M, FitzGerald LM, Vezzuli S, Reid J, Malacarne G, Iliev D, Coppola G, Wardell B, Micheletti D, Macalma T, Facci M, Mitchell JT, Perassolli M, Eldredge G, Gatto P, Oyzerski R, Moretto M, Gutin N, Stefanini M, Chen Y, Segala C, Davenport C, Dematte L, Mraz A, Battilana J, Stormo K, Costa F, Tao Q, Si-Ammour A, Harkins T, Lackey A, Perbost C, Taillon B, Stella A, Solovyev V, Fawcett JA, Sterck L, Vandepoele SK, Fontana P, Gutin A, Ven De Peer Y, Salamini F, Viola R (2007). **A high**-**quality draft concensus sequence of the genome of a heterozygous grapevine variety**. PLoS One.

[30] Schnable PS (2009). The B73 maize genome: complexity, diversity, and dynamics. Science.

[31] Andrews S: *FastQC: A Quality Control Tool for High Throughput Sequence Data*; 2010. http://www.bioinformatics.babraham.ac.uk/projects/fastqc.

[32] Goecks J, Nekrutenko A, Taylor J (2010). Galaxy: a comprehensive approach for supporting accessible, reproducible, and transparent computational research in the life sciences. Genome Biol.

[33] Giardine B, Riemer C, Hardison RC, Burhans R, Elnitski L, Shah P, Zhang Y, Blankenberg D, Albert I, Taylor J, Miller W, Kent WJ, Nekrutenko A (2005). **Galaxy**: **a platform for interactive large**-**scale genome analysis**. Genome Res.

[34] Blankenberg D, Von Kuster G, Coraor N, Ananda G, Lazarus R, Mangan M, Nekrutenko A, Taylor J (2010). **Galaxy**: **a web**-**based genome analysis tool for experimentalists**. Curr Protoc Mol Biol.

[35] Joshi NA, Fass JN: **Sickle: A Sliding-Window, Adaptive, Quality-Based Trimming Tool for FastQ Files (Version 1.21)** *[Software]*; 2011. Available at https://github.com/najoshi/sickle.

[36] Li H, Durbin R (2010). Fast and accurate long-read alignment with Burrows-Wheeler transform. Bioinformatics.

[37] Li H, Handsaker B, Wysoker A, Fennell T, Ruan J, Homer N, Marth G, Abecasis G, Durbin R (2009). The Sequence Alignment/Map format and SAMtools. Bioinformatics.

[38] Glazebrook J, Drenkard E, Preuss D, Ausubel FM (1998). Use of cleaved amplified polymorphic sequences (CAPS) as genetic markers in Arabidopsis thaliana. Methods Mol Biol.

[39] Tucker EJ, Huynh BL (2014). Genotyping by high-resolution melting analysis. Methods Mol Biol.

[40] Fawcelt T (2006). An introduction to ROC analysis. Pattern Recogn Lett.

