# Supplementary material for: Mapping mutations in plant genomes with the user-friendly web application CandiSNP
Source: Plant Methods. 2014 Dec 30;10:41. doi: 10.1186/s13007-014-0041-7 (PMC4301057; doi:10.1186/s13007-014-0041-7)
Supplement: Additional file 1: — Calculation of expected SNP frequencies in out- and back-crosses. SNP frequencies are calculated from different types of crosses using available data. [file 13007_2014_41_MOESM1_ESM.pdf]

Additional File 1: Calculation of expected SNP frequencies in out- and back-crosses.

Graham J Etherington[1,2†], Jacqueline Monaghan[1†], Cyril Zipfel[1] and Dan MacLean[1*]

[*]Correspondence:
dan.maclean@tsl.ac.uk
[1]The Sainsbury Laboratory, Norwich Research Park, NR4 7UH, Norwich, UK
Full list of author information is available at the end of the article
[†]Equal contributor

SNP frequency in different crosses

Methods such as SHOREMap [1] and NGM [2] rely on the relative density of SNPs of one type to SNPs of another type to work (usually the number of homozygous SNPs relative to heterozygous SNPs). As a second ecotype is involved in an out-cross then you get many thousands of SNPs introduced. We can define some terms

- S_{ler} the number of SNPs between the reference sequence and another ecotype (e.g Ler)
- S_{ref} the number of SNPs between the reference sequence (i.e Col-0) and the bulked mutants (i.e *bak1-5 mob1* and *bak1-5 mob2*).
- S_{parent} the number of SNPs present in the parent (i.e in *bak1-5*)
- S_{mut} the number of SNPs present in the mutant

The formula for the number of SNPs in an out-cross is

$$S_{\text{ref}} \cup S_{\text{parent}} \cup S_{\text{ler}} \tag{1}$$

(where the symbol $\cup$ is the symbol that defines the 'union' operation, the joining of two sets with removal of overlaps). So this formula represents the non-redundant, summed set of the SNPs in each component of the background. Looking at the relative magnitudes of the numbers in each of those subsets of SNPs

- S_{ref} is on the order of 1200 [3]
- S_{parent} is on the order of 500 [4]
- S_{mut} is on the order of 500 for the same reasons as above
- S_{ler} is on the order of 150,000 [1, 3]

So in the Arabidopsis genome we would expect 1 SNP every 900 nt in a out-crossed experiment with another ecotype and 1 SNP every 65,000 nt in the back-cross.

We see then that any cross with another ecotype is going to be more amenable to statistical methods. The number of SNPs from a backcross is not amenable to frequentist approaches over an entire genome.

Author details
[1]The Sainsbury Laboratory, Norwich Research Park, NR4 7UH, Norwich, UK. [2]The Genome Analysis Centre Norwich Research Park, NR4 7UH, Norwich, UK.

References

1. Schneeberger, K., Ossowski, S., Lanz, C., Juul, T., Petersen, A.H., Nielsen, K.L., Jørgensen, J.-E., Weigel, D., Andersen, S.U.: SHOREmap: simultaneous mapping and mutation identification by deep sequencing. Nature methods **6**(8), 550–551 (2009)
2. Austin, R.S., Vidaurre, D., Stamatiou, G., Breit, R., Provart, N.J., Bonetta, D., Zhang, J., Fung, P., Gong, Y., Wang, P.W., McCourt, P., Guttman, D.S.: Next-generation mapping of Arabidopsis genes. The Plant journal : for cell and molecular biology **67**(4), 715–725 (2011)
3. Ossowski, S., Schneeberger, K., Clark, R.M., Lanz, C., Warthmann, N., Weigel, D.: Sequencing of natural strains of Arabidopsis thaliana with short reads. Genome research **18**(12), 2024–2033 (2008)
4. Weigel, D., Glazebrook, J.: EMS Mutagenesis of Arabidopsis Seed. Audio and Electroacoustics Newsletter, IEEE **2006**(5), (2006)
