# Supplementary material for: Mapping mutations in plant genomes with the user-friendly web application CandiSNP
Source: Plant Methods. 2014 Dec 30;10:41. doi: 10.1186/s13007-014-0041-7 (PMC4301057; doi:10.1186/s13007-014-0041-7)
Supplement: Additional file 2: — SNP calling script. To create our CandiSNP input files we used the pileups_to_snps.rb Ruby script which relies on the bio-samtools and bio-gngm Ruby Gems. The input data is the FASTA reference sequence (TAIR10 genome in this case) and the SAM/BAM alignment file for each dataset. The output is a comma-delimited file of SNPs formatted for input to the CandiSNP application. The source code for this script is available at https://github.com/danmaclean/candisnp/blob/master/pileup_to_snps.rb. [file 13007_2014_41_MOESM2_ESM.pdf]

```ruby
#!/usr/bin/env ruby
#
#  untitled
#
#  Created by Dan MacLean (TSL) on 2013-01-09.
#  Copyright (c)  . All rights reserved.
###################################################
require 'rubygems'
require 'bio-samtools'
require 'bio-gngm'

sequences = Bio::DB::FastaLengthDB.new(:file => ARGV[0])

bam = Bio::DB::Sam.new(:bam=>ARGV[1], :fasta=>ARGV[0])
bam.open
puts('"Chr","Pos","Ref","Alt","Allele_Freq"')
sequences.each do |id,length|
   $stderr.puts "on #{id}:1-#{length} "
   bam.mpileup(:r => "#{id}:1-#{length}", :Q => 20, :q => 20) do |pileup|
     #puts "Consensus: " + pileup.consensus
     if pileup.is_snp?(:ignore_reference_n => true, :min_depth => 6, :min_non_ref_count => 3) and pileup.consensus != pileup.ref_ba
       mut = "FALSE"
       mut = "TRUE" if (pileup.ref_base == 'G' and pileup.consensus == 'A') or (pileup.ref_base == 'C' and pileup.consensus == 'T')
       puts [pileup.ref_name, pileup.pos, pileup.ref_base, pileup.consensus, pileup.non_ref_count / pileup.coverage, mut ].join(","
     end
   end
end
```

Additional File 2: SNP calling script.

To create our CandiSNP input files we used the pileups_to_snps.rb Ruby script which relies on the bio-samtools and bio-gngm Ruby Gems. The input data is the FASTA reference sequence (TAIR10 genome in this case) and the SAM/BAM alignment file for each dataset. The output is a comma-delimited file of SNPs formatted for input to the CandiSNP application. The source code for this script is available at:
https://github.com/danmaclean/candisnp/blob/master/pileup_to_snps.rb
