# Supplementary material for: Mapping mutations in plant genomes with the user-friendly web application CandiSNP
Source: Plant Methods. 2014 Dec 30;10:41. doi: 10.1186/s13007-014-0041-7 (PMC4301057; doi:10.1186/s13007-014-0041-7)
Supplement: Additional file 3: — Simplifying SNP analysis by comparing multiple mutant genomes. Comparing multiple mutant genomes identifies unique SNPs in each mutant. [file 13007_2014_41_MOESM3_ESM.pdf]

Additional File 3: Simplifying SNP analysis by comparing multiple mutant genomes.

Graham J Etherington[1,2†], Jacqueline Monaghan[1†], Cyril Zipfel[1] and Dan MacLean[1*]

[*]Correspondence:
dan.maclean@tsl.ac.uk
[1]The Sainsbury Laboratory, Norwich Research Park, Norwich, UK
Full list of author information is available at the end of the article
[†]Equal contributor

Categorising SNP accumulation in Arabidopsis

SNPs should come from three places:

- S_{ref} the number of SNPs between the reference sequence (i.e. Col-0) and the bulked mutants (i.e *bak1-5 mob1* and *bak1-5 mob2*).
- S_{parent} the number of SNPs present in the parent (i.e in *bak1-5*)
- S_{mut} the number of SNPs present in the mutant

The set of SNPs in the mutant relative to the Col-0 reference S_{tot} is the set made from combining all these SNPs (not counting any SNP twice if it is in the same nucleotide from a different background).

$$S_{\mathrm{tot}} = S_{\mathrm{ref}} \cup S_{\mathrm{parent}} \cup S_{\mathrm{sup}} \quad (1)$$

The number of elements in this set is referred to as $|S_{\mathrm{tot}}|$.

The number of SNPs in the set (i.e in a *mob* mutant) that can be called in an NGS experiment is C, the number of actual SNPs multiplied by the probability of calling a SNP (i.e the accuracy of the NGS experiment).

$$C = |S_{\mathrm{tot}}| \times p(\text{calling a SNP in an NGS experiment}) \quad (2)$$

For completeness the number of SNPs missed is

$$|S_{\mathrm{tot}}| - C \quad (3)$$

What power is gained (or lost) by having more mutants?

The screen in our hands was empowered somewhat by having more than one mutant, allowing us to delete SNPs that were called in more than one experiment and are therefore not unique and, presumably not those introduced by the mutagenesis. SNP analysis pipelines are not absolutely accurate and not all SNPs present in the genomes will be called in a given SNP analysis. Ommisions and false SNP calls can occur due to issues with sequence coverage, errors in the sequence reads, errors by alignment programs or other bioinformatics issues. If we consider a perfect experiment in which we don't miss any SNPs then the increase in power from having more mutants is the proportion that we can reduce the non-mutant related SNPs in each. This is just the overlap between the two sets of SNPs, labelled earlier as S_{ref} and S_{parent} and is on the order of 1700 [1, 2] ($S_{\mathrm{ref}} + S_{\mathrm{parent}}$, see Additional File 1) . As the Arabidopsis genome is 130 millions of nucleotides long then the chance of two

independent SNP lists overlapping is clearly very small, and the worst that could happen is you get 1700 extra SNPs that aren't related to ones in other mutants. Of course, these mutants are very closely related so they are not independent and the other extreme case is that all the SNPs would be shared. Two important factors are the amount to which they are related and the ability to call them correctly, the last quantity was noted earlier as C. Let's consider the extreme case where the S_{ref} SNPs are completely related between the two mutants.

The proportion of SNPs missed the first time round m is basically 1 minus the probability we will pick up the SNP

$$m = 1 - Prob(\text{calling a SNP in an NGS experiment}) \tag{4}$$

In the second round with another mutant, then the new proportion missed, m_2 is the proportion we missed in the first round, m, minus the proportion that we get this time. The proportion missed overall is the difference between those missed in the first mutant, minus those picked up in the second mutant. So if we missed 0.05 the first time, and call SNPs with 0.95 accuracy, then the maximum proportion that can be picked up is 0.05×0.95. Putting these together,

$$m_2 = m - [m \times Prob(\text{calling a SNP in an NGS experiment})] \tag{5}$$

and for further mutants, its just repetition of the same pattern. So for a third mutant the number of SNPs missed m_3

$$m_3 = m_2 - [m_2 \times Prob(\text{calling a SNP in an NGS experiment})] \tag{6}$$

So now we see a pattern emerging for the expression given certain numbers of mutants. If we want to check a little further it makes sense to replace that long term with a short alternative p

$$p = Prob(\text{calling a SNP in an NGS experiment}) \tag{7}$$

And we redo the whole thing with p. Here we'll rename m to keep it meaning literally the proportion missed, and introduce q which is $1 - p$.

$$p = Prob(\text{calling a SNP in an NGS experiment})$$
$$q = 1 - p$$

$$m_1 = q - pq$$
$$m_2 = (q - pq) - pq$$
$$m_3 = ((q - pq) - pq) - pq)$$

Then with a little algebra, we can simplify each term by taking out the factor q

$$\begin{aligned}
m_1 &= q - pq \\
&= q(1-p) \\
m_2 &= (q - pq) - pq \\
&= q(1-p) - pq \\
m_3 &= ((q - pq) - pq) - pq) \\
&= q(1-p) - pq - pq
\end{aligned}$$

So the pattern again is that we just end up adding a $-pq$ to the end, so that for any number (a) of mutants, the proportion missed m_a is

$$m_a = q(1-p) - pq(a-1) \tag{8}$$
$$m_a = q^2 - pq(a-1) \tag{9}$$

Note that this is for the extreme case, where the SNPs not due to the mutagenesis are completely shared. We can now look at how not sharing complete sets of non-mutagenesis induced SNPs affects this proportion, a situation like that when we sequence bulks, for example.

What if the SNP sets are not completely shared?

One assumption of the model so far is that the natural SNPs are completely shared due to the plants being closely related. Its worth noting that when the SNPs don't overlap we can't remove them, that is they're disjoint sets. We can model the unique parts of each set by simply adding back in the proportion that can't be reached o that represents the non-overlapping proportion (so is a number between 0 and 1) we get a new, but very similar, model.

$$m_a = q^2 - pq(a-1) + o \tag{10}$$

We could be more clever with o, for example making it a function rather than a constant, so perhaps making it the result of a sum calculating the probability of overlap given the numbers, but for these purposes the proportion should suffice.

So the model shows that with a pipeline that has a non-negligible chance of missing SNPs then adding extra mutants works for maybe one or two more, but doesn't really make a difference after that.

Visualising this relationship

Naturally, one would want to see the relationship graphed. There are just two variables: the number of mutants and the proportion of SNPs not called (the accuracy of the SNP calling pipeline, not the same as the number of SNPs called that are

accurate). Lets first look at a range of figures for the accuracy of the SNP calling pipeline from 0.99 to 0.5, for 1 to 5 mutants with complete overlap in the SNPs. Here's how the graph looks (Figure 1)

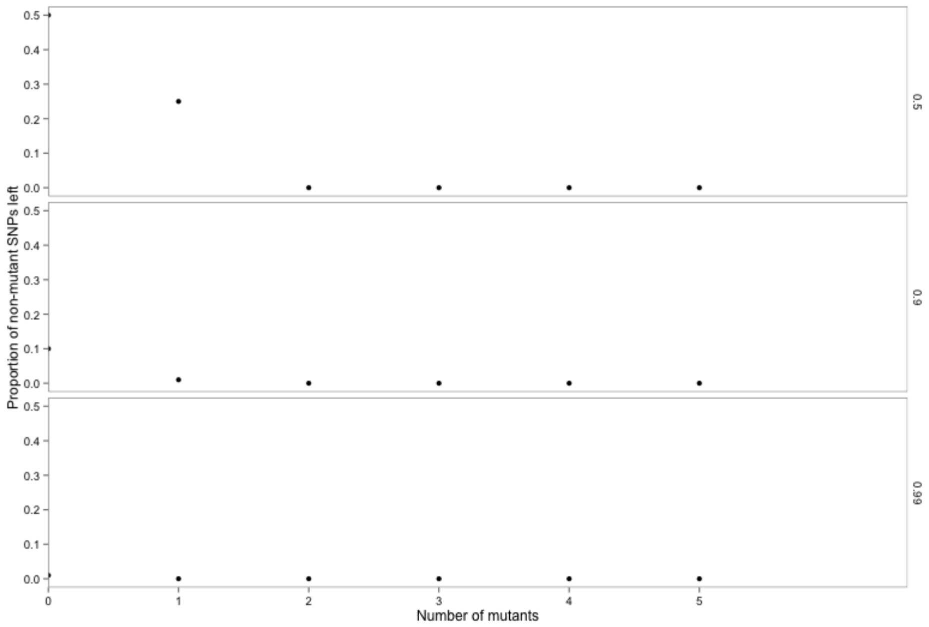

Figure 1 Simulated power of using extra mutant lines to remove non mutagenesis SNPs. Values from model, proportion of SNPs missed by addition of extra mutants with o constant, proportion of SNPs missed by addition of extra mutants at $Prob$(calling a SNP in an NGS experiment) $= p = 0.5, 0.9$ and 0.99.

The basic pattern is that adding extra mutants (more than 3, say) doesn't improve things very much at all (Except in $p = 0.99$ where it doesn't make any difference because we get all the SNPs the first time).

What happens in the real data?

The main question really after that exercise is what dynamic does the real data show. Because we have multiple mutants we can check it out. To do this we took the permutations of the two mutants and worked out the reduction in number of SNPs by deleting the overlapping SNPs from the other mutant, for the two permutations of two mutants. These are plotted in Figure 2. The figure shows number of SNPs rather than proportion as in the model, but the dynamic is very similar. The increase in power is marked even with just one extra mutant. The number of SNPs that are different between a parent line and mutant would be simply the number of SNPs introduced by the mutagenesis and the natural variation between the plants themselves. It is therefore the same as having a single mutant

We have then a basic model for the relationship and a tool for examining how many extra mutants a researcher would need to maximise their chances of getting down to the truly unique SNPs in each mutant line.

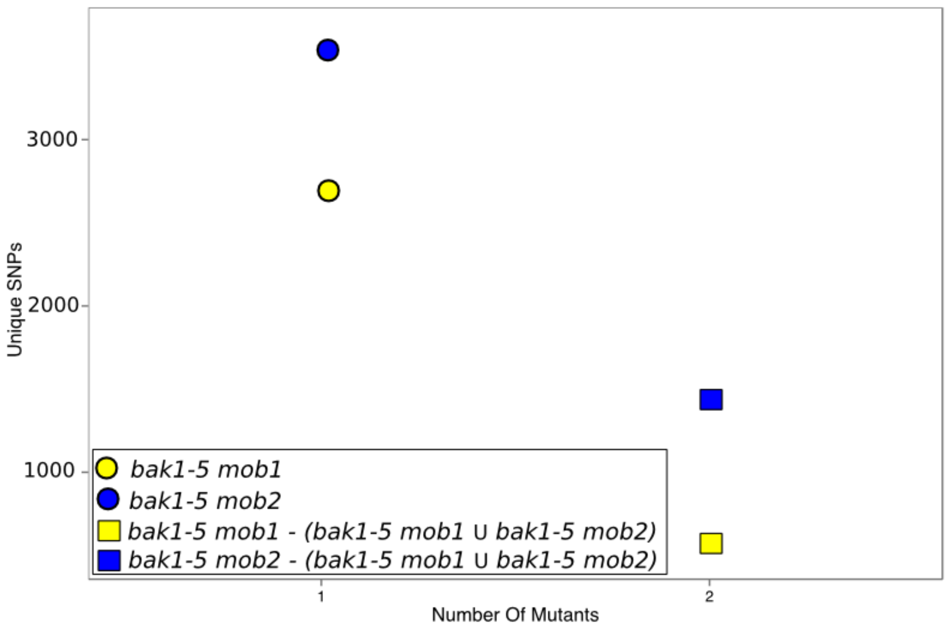

Figure 2 Experimental demonstration of power of using one extra mutant lines to remove non mutagenesis SNPs. Values from real data for *bak1-5 mob1* and *bak1-5 mob2*. The plot demonstrates the number of SNPs left after removing those shared between the two mutants (noted as *bak1-5 mob1* ∪ *bak1-5 mob2*).

Author details
[1]The Sainsbury Laboratory, Norwich Research Park, Norwich, UK. [2]The Genome Analysis Centre, Norwich Research Park, Norwich, UK.

References

1. Ossowski, S., Schneeberger, K., Clark, R.M., Lanz, C., Warthmann, N., Weigel, D.: Sequencing of natural strains of Arabidopsis thaliana with short reads. Genome research **18**(12), 2024–2033 (2008)
2. Weigel, D., Glazebrook, J.: EMS Mutagenesis of Arabidopsis Seed. Audio and Electroacoustics Newsletter, IEEE **2006**(5), (2006)
