# Supplementary material for: Mapping mutations in plant genomes with the user-friendly web application CandiSNP
Source: Plant Methods. 2014 Dec 30;10:41. doi: 10.1186/s13007-014-0041-7 (PMC4301057; doi:10.1186/s13007-014-0041-7)
Supplement: Additional file 5: — Genome-wide SNP density plots for bak1 - 5 mob2. Using the ‘CandiSNP palette’, all SNPs with allele frequencies >75% are plotted in grey, while candidate causative SNPs (defined as those causing non-synonymous changes in gene-coding regions) are plotted in red. These plots are also available at http://dx.doi.org/10.6084/m9.figshare.1250027. [file 13007_2014_41_MOESM5_ESM.pdf]

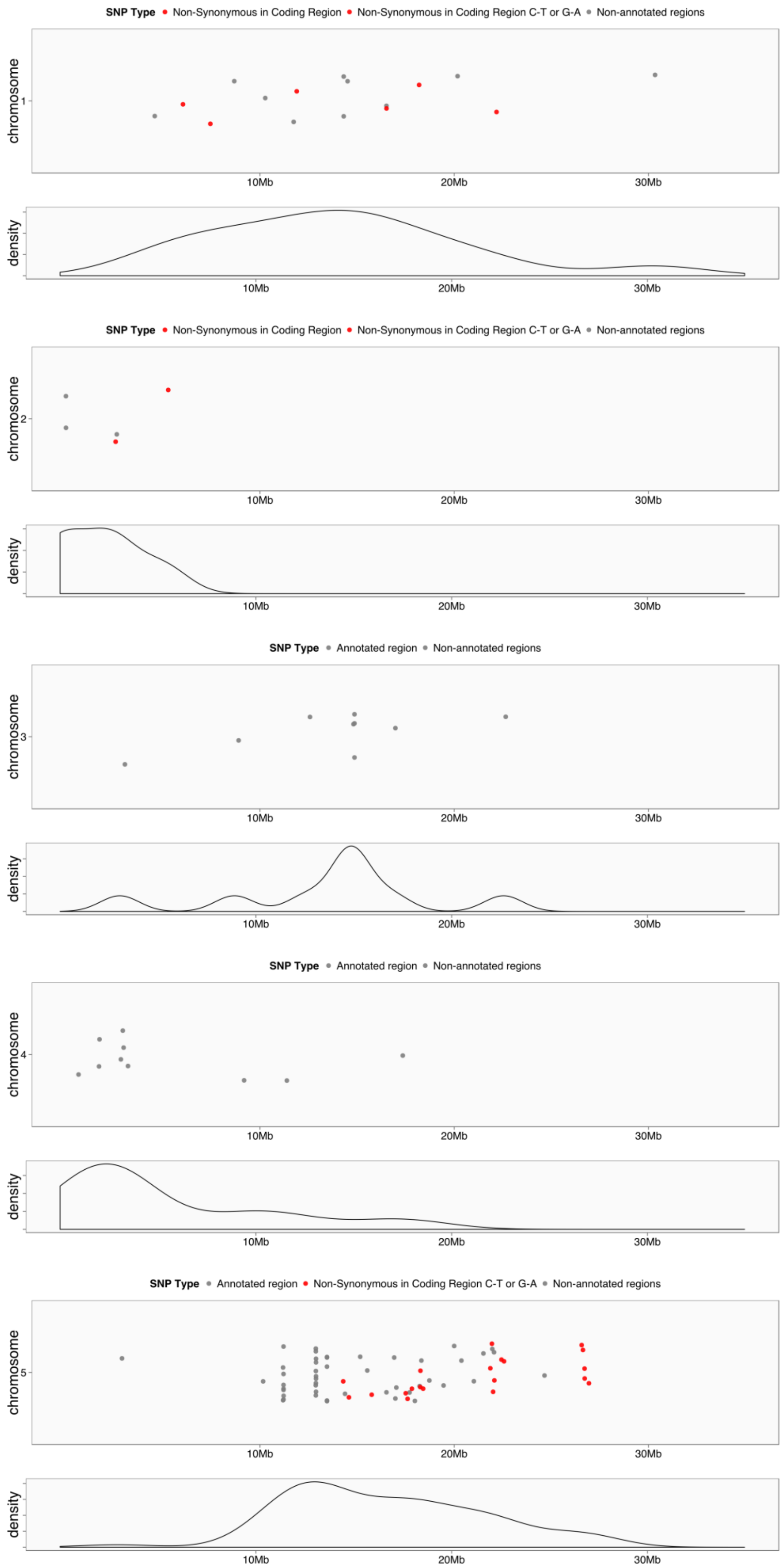
SNP Type • Non-Synonymous in Coding Region • Non-Synonymous in Coding Region C-T or G-A • Non-annotated regions
chromosome
1
10Mb
20Mb
30Mb
density
10Mb
20Mb
30Mb
SNP Type • Non-Synonymous in Coding Region • Non-Synonymous in Coding Region C-T or G-A • Non-annotated regions
chromosome
2
10Mb
20Mb
30Mb
density
10Mb
20Mb
30Mb
SNP Type • Annotated region • Non-annotated regions
chromosome
3
10Mb
20Mb
30Mb
density
10Mb
20Mb
30Mb
SNP Type • Annotated region • Non-annotated regions
chromosome
4
10Mb
20Mb
30Mb
density
10Mb
20Mb
30Mb
SNP Type • Annotated region • Non-Synonymous in Coding Region C-T or G-A • Non-annotated regions
chromosome
5
10Mb
20Mb
30Mb
density
10Mb
20Mb
30Mb

**Additional File 5: Genome-wide SNP density plots for *bak1-5 mob2.***

All SNPs with allele frequencies >75% are plotted in grey, while candidate causative SNPs (defined as those causing non-synonymous changes in gene-coding regions) are plotted in red. These plots are also available at http://dx.doi.org/10.6084/m9.figshare.1250027.
