# Supplementary material for: Mapping mutations in plant genomes with the user-friendly web application CandiSNP
Source: Plant Methods. 2014 Dec 30;10:41. doi: 10.1186/s13007-014-0041-7 (PMC4301057; doi:10.1186/s13007-014-0041-7)
Supplement: Additional file 6: — Primers used in this study. Genomic regions containing candidate SNPs in bak1-5 mob1 and bak1-5 mob2 were amplified by PCR using these primers and Sanger-sequenced alongside bak1-5 as a control. [file 13007_2014_41_MOESM6_ESM.pdf]

bak1-5 mob1

SNP location	Primer sequence (5'-3')
I_825457_F	TGTGAATAGAATGGACTCTC
I_825457_R	TGCCATAGGATCTCTCACTT
I_11892068/070/252_F	TCTTCTCGTCCCACATATC
I_11892068/070/252_R	AACAAGGTGATGAAGAAATGGA
I_16516501_F	GATCGGGGTTCCTCAAAGT
I_16516501_F	CACAAGATGATGTTCATAAAGT
I_16525522_F	CTCATCAGTACTATAGTCCTC
I_16525522_F	TGTTCACTAGGACCCTTAGTGT
I_24243231_F	TGCAGAGATGTCTACCACGG
I_24243231_R	TATCACTATGAGTGACACTTA
V_23206476_F	ACACATTTGACCGCTGAATCAC
V_23206476_R	TGTGATTGCTTATATAATCATC
V_26457834_F	AGATTCGCCTCTAAAGGCTA
V_26457834_R	TTCTCTAACCCACGCATGTG
V_26458077_F	AGATTCGCCTCTAAAGGCTA
V_26458077_R	TTCTCTAACCCACGCATGTG
V_26474069_F	CGTTGAGAACTTGATCAATG
V_26474069_R	GAGGATCTGTTTCAGTTCGC

bak1-5 mob2

SNP location	Primer sequence (5'-3')
I_7446564_F	AGAAAATTAAGCACCGGA
I_7446564_R	TGTGTTGTAATATTCTGG
I_11892984_F	TCTGCAACATCATTATATTCATCATGT
I_11892984_F	TTAGGCATCGAAAGAGTTCC
I_16513961_F	TGATCGGACTCAGCATAATCCT
I_16513961_F	CTACAAGTCATCTAGGGCCAA
I_17757465_F	TGCTGCGACTCGGTATTC
I_17757465_R	CCAGAATTCATAAACAAT
I_18192647_F	GAGTTCGGCCAACTCAATGAC
I_18192647_R	TTGCAAGCATTCGACAAGTACCT
I_22178447_F	TGTTCAGCCGCTATTCCAAGA
I_22178447_R	TAGTATGAATAAGTCTTTGG
II_2568811_F	TGCCCTTTCAACCAATTCGG
II_2568811_R	TCGCAGATGGGTAAGAGCAGC
IV_2362567_F	ACGGTTTACTAACCAATTAC
IV_2362567_R	TGGAAGGGTGCAAGGAACGGATG
V_5569896_F	GTGATATATGAGGAACAGAAG
V_5569896_R	AGATCTTCGAGCCAGCTCTCGGG
V_14285189_F	CTGGTTGCCAAACATAAC
V_14285189_R	GGAATGACGACTCAGGACGGAGCTG
V_15751875_F	TGGGTATTATAGGAATGGTT
V_15751875_R	ATTGCACTCCAAGGACCGGTATGG
V_17503318_F	TGCACCAGGCATAATT
V_17503318_R	ATTTCAGCTGAACTATCTAC

V_17820568_F<br>V_17820568_R	TCTGACAGCAAAGCTGGAGAT<br>TAGATTCAAACTAATCATACT
V_18251689_F<br>V_18251689_R	CGTTAATTAATTTGTAGT<br>CCTTAGCTGCTTCTGCTCCTT
V_18261108_F<br>V_18261108_R	CTTGCTGACACTTTAGAGTC<br>CTCATCAATAACCACCTCTTCTTG
V_18399206_F<br>V_18399206_R	ATGGCGGTGAGTTTGACGG<br>TCGGAACTGTTATGAACAACTTG
V_21859555_F<br>V_21859555_R	CTGCATGGAGTGTCTGGCGAA<br>CATTAGACATGGTACTATCA
V_21939106_F<br>V_21939106_R	GACTTTGTGTGCCACTACTTTA<br>TGGCCAACATTTTGCATCCACAG
V_22002355_F<br>V_22002355_R	ATATTGACCAGCAATAGCTT<br>GACCATTGGAACGCCGCCATTCG
V_22066915_F<br>V_22066915_R	GATTTCATACCTTAAATTAGTG<br>ATATCATCAGTTGATAAAGCAT
V_22430866_F<br>V_22430866_R	CTTCTCAATGGGGACATCATG<br>GAGACGGTGACGTTCCTG
V_22449787_F<br>V_22449787_R	GGCGGAGGAACAAGGTTCC<br>AAAGGATTGGATGACT
V_22565056_F<br>V_22565056_R	GACAATAAAAGCATTAGCTAA<br>CTCTAAGTCATCATCATGTTGT
V_26458017_F<br>V_26458017_R	AGATTCGCCTCTAAAGGCTA<br>TTCTCTAACCCACGCATGTG
V_26560691_F<br>V_26560691_R	CAAGTTTCTAACAAGAAC<br>CTGATTCTGGGTTCTCA
V_26626055_F<br>V_26626055_R	CCCGACGAACCGGTGTACCG<br>TCCACCTAGAAATATCCTCCTTT
V_26710709_F<br>V_26710709_R	GGTTATAGTTTCTTAGGAAG<br>TCCCATAGTTACAAGTCATTCAC
V_26716839_F<br>V_26716839_R	CGTTTCAATATCTGCAATACT<br>TGTGGTTACTCTTGTGGTT
V_26935248_F<br>V_26935248_R	TCAGGAGCTAGGCCTAACATG<br>CCACGCCGGAGGCACTGTAAG

Additional File 6: Primers used in this study.

Genomic regions containing candidate SNPs in *bak1-5 mob1* and *bak1-5 mob2* were amplified by PCR using these primers and Sanger-sequenced alongside *bak1-5* as a control.
