# Supplementary material for: Mapping mutations in plant genomes with the user-friendly web application CandiSNP
Source: Plant Methods. 2014 Dec 30;10:41. doi: 10.1186/s13007-014-0041-7 (PMC4301057; doi:10.1186/s13007-014-0041-7)
Supplement: Additional file 7: — Receiver operating characteristic curve plot. Receiver operating characteristic curve plot, demonstrating the optimal parameters for use in CandiSNP on bak1-5 mob1. The proportion of false positives (non-causative SNPs identified by CandiSNP regardless of location and category) is also plotted as a comparison to the standard sensitivity and specificity [40]. The overlaid dashed line represents the optimal point that maximises the ability of CandiSNP to find real causative SNPs (sensitivity) while minimising the inclusion of false positives. [file 13007_2014_41_MOESM7_ESM.pdf]

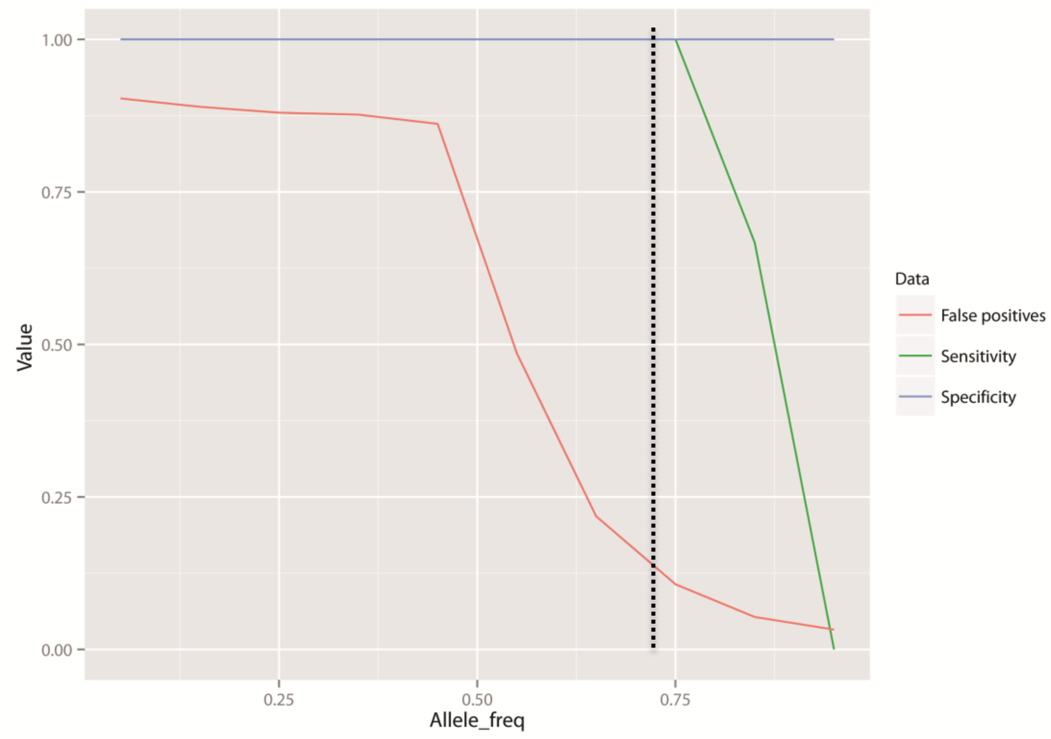

Additional File 7: Receiver Operating Characteristic curve plot.

Receiver operating characteristic curve plot, demonstrating the optimal parameters for use in CandiSNP on *bak1-5 mob1*. The proportion of false positives (non-causative SNP identified by CandiSNP regardless of location and category) is also plotted as a comparison to the standard sensitivity and specificity [40]. The overlaid dashed line represents the optimal point that maximises the ability of CandiSNP to find real causative SNPs (sensitivity) while minimising the inclusion of false positives.
